# Cold atmospheric plasma-aerosol treatment of equine dermatophytosis: a novel therapeutic approach

**DOI:** 10.3389/fvets.2025.1651946

**Published:** 2025-07-28

**Authors:** Sandra Kurras, Derek Knottenbelt, Ulrich Schmelz, Tom Schaal, Tim Tischendorf, Robert Fuchs, Timon Schorling, Marc H. W. Koene

**Affiliations:** ^1^Tierklinik Lüsche GmbH, Bakum, Germany; ^2^Equine Medical Solutions Ltd, Stirling, Scotland, United Kingdom; ^3^Medical Microbiology, Drinking Water and Hygiene Laboratory, Hygiene and Medical Microbiology, Universitätsmedizin Göttingen, Fulda, Germany; ^4^Faculty of Health and Healthcare Sciences, University of Applied Sciences Zwickau, Zwickau, Germany; ^5^Research and Development, WK-MedTec GmbH, Bückeburg, Germany

**Keywords:** cold atmospheric plasma treatment, equine dermatophytosis, antifungal treatment, *Trichophyton mentagrophytes*, *Trichophyton benhamiae*, *Trichophyton tonsurans*

## Abstract

**Introduction:**

Dermatophytosis is a common fungal skin infection in horses, particularly affecting young and immunocompromised animals. Traditional treatments often involve antifungal medications with potential side effects. Here, we present a case report that evaluated the efficacy of cold atmospheric plasma-aerosol (CAP-A) as a standalone treatment for equine dermatophytosis.

**Methods:**

A 2-year-old Westphalian warmblood stallion presented with confirmed dermatophyte lesions restricted to the left side of the mouth which were treated with CAP-A. Treatment was administered 2 times daily for 12 consecutive days; each session consisted of two 3-min fogging cycles. Microbiological samples were collected before and after the treatment period. Daily photographic documentation was maintained.

**Results:**

Initial microbiological examination identified *Trichophyton* ssp. (*Trichophyton mentagrophytes, Trichophyton benhamiae, Trichphyton erinacei, Trichophyton tonsurans, Trichophyton equinum, Trichophyton verrucosum,* or *Trichophyton rubrum*) as the definitive aetiology. Post-treatment samples were negative for all of the tested dermatophytes including *Trichophyton* spp., *Microsporum canis*, and *Nannizzia* ssp. Clinical progression was documented through photographic evidence. The horse showed no signs of discomfort during or after the treatment sessions.

**Discussion:**

CAP-A therapy demonstrated promising results as a non-pharmacological treatment option for equine dermatophytosis, achieving both clinical and microbiological resolution without adverse effects. This single case report will need to be followed up by a prospective study in a larger sample to draw definite conclusions about the efficacy of the treatment.

## 1 Introduction

Dermatophytosis, predominantly due to *Trichophyton* spp. (*Trichophyton mentagrophytes, Trichophyton benhamiae, Trichphyton erinacei, Trichophyton tonsurans, Trichophyton equinum, Trichophyton verrucosum,* or *Trichophyton rubrum*), particularly affects young horses and represents a significant and persistent clinical challenge in equine practice. The spores of all significant dermatophytes are strongly resistant to environmental challenges; and immunity to infections in affected patients is variable. Current treatment protocols typically involve topical medicated washes and/or systemic antifungal medications, which may cause unwanted side effects and patient discomfort and, in any case, require prolonged and/or repeated treatment courses (1–4).

**Figure 1:**
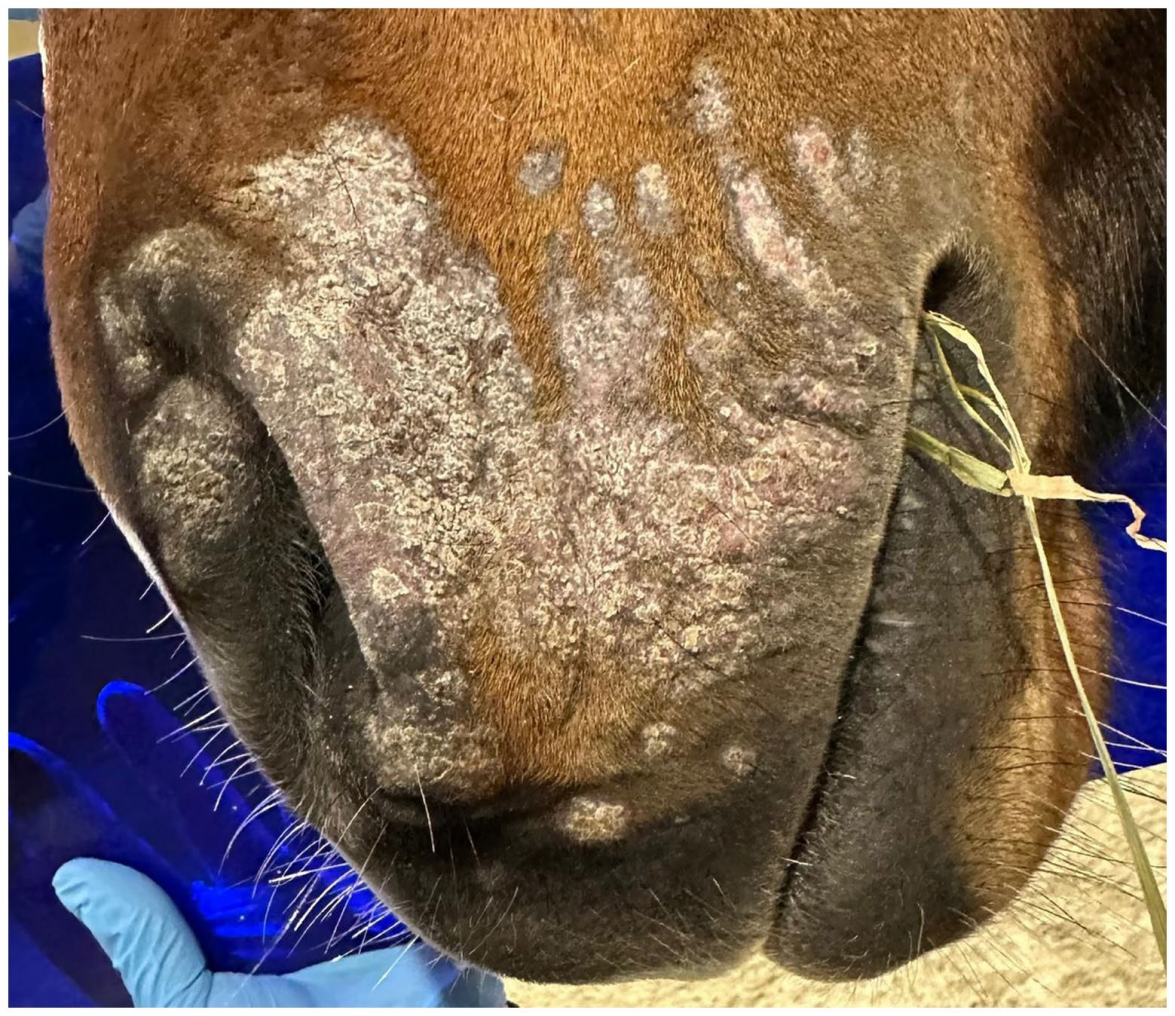
PreTreatment_Lateral.

**Figure 2:**
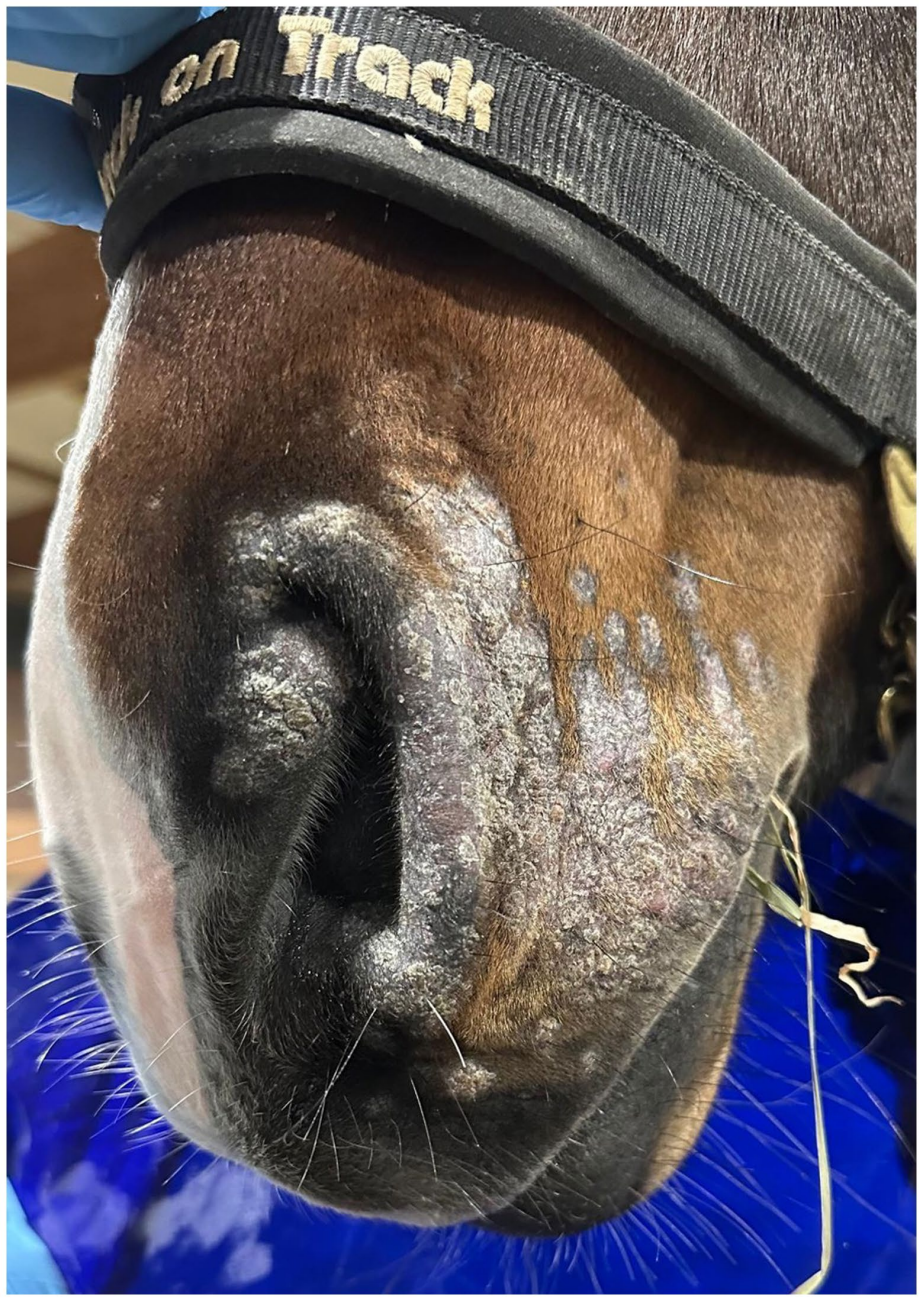
PreTreatment_Frontal.

**Figure 3:**
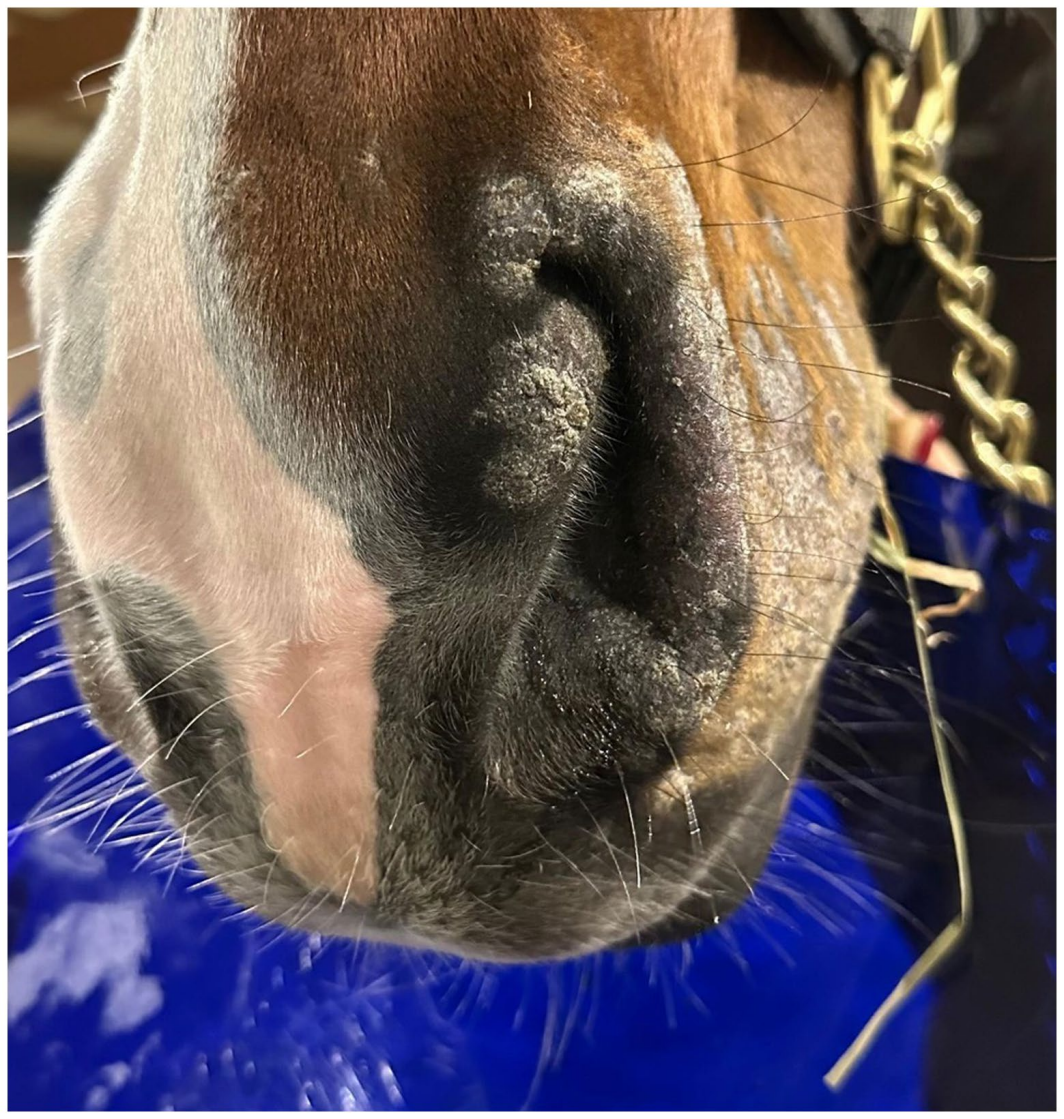
PreTreatment_Oblique.

Cold atmospheric plasma-aerosol (CAP-A) technology offers a novel therapeutic approach through indirect application of cold atmospheric plasma products (Reactive Oxygen Species—ROS) that selectively target microorganisms and damage tissue while sparing healthy cells (5, 6). Here, we report the case of a 2-year-old Westphalian warmblood stallion presented with characteristic dermatophyte lesions apparently restricted to the left side of the face. No previous treatment had been administered. Initial examination revealed typical, nonpainful, non-pruritic, roughly circular, alopecic, scaling ‘ringworm’ lesions (Figures 1–3). Skin scrapings and swabs were collected for mycological culture. Laboratory analysis (Antech Lab, Germany) confirmed the presence of *Trichophyton* ssp. (*Trichophyton mentagrophytes, Trichophyton benhamiae, Trichphyton erinacei, Trichophyton tonsurans, Trichophyton equinum, Trichophyton verrucosum,* or *Trichophyton rubrum*).

## 2 Methods

The horse received CAP-A therapy using a specialized delivery device (PLASMO®VET, WK-Medtec GmbH, Bückeburg, Germany) that generates plasma-activated aerosol through patented cold atmospheric plasma technology.

The PLASMO®VET system operates as a non-thermal plasma generator requiring standard electrical supply with stabilized operating water. The system generates cold atmospheric plasma (CAP) through dielectric barrier discharge technology operating at atmospheric pressure. Thereafter, the cold plasma reaction products condition and enrich nebulized water (aerosol) obtaining cold atmospheric plasma-aerosol (CAP-A).

Technical specifications include:

- Operating voltage: 1450–1750 V at high frequency (38 k Hz).
- Gas composition: Ambient air with controlled humidity.
- Plasma temperature: <40°C (non-thermal).
- Active species generated *in situ*: reactive oxygen species, e.g., Hydroxyl radicals (OH), superoxide anions (O_2_), hydrogen peroxide (H_2_O_2_), ozone (O_3_), and singlet oxygen (O=O).
- Aerosol particle size: 0.1–5 μm for optimal tissue penetration.

The device offers several clinical advantages including pain-free, noise-free operation enabling stress-free application in equine patients, large area coverage for efficient treatment of extensive lesions, and immediate availability without preparation time. The technology demonstrates excellent safety profile with no thermal tissue damage risk, no systemic absorption concerns, and no special protective equipment requirements for operators.

Therapy of CAP-A exerts its antimicrobial effects through multiple synergistic mechanisms targeting dermatophyte pathogens. The principal mechanism of action is of physical nature, the solubility and displacement effect. Further, the plasma generates a complex mixture of reactive oxygen species, including hydroxyl radicals (•OH), superoxide anions (O_2_•-), hydrogen peroxide (H_2_O_2_), ozone (O_3_), and singlet oxygen (O=O), which collectively create a hostile environment for fungal survival. These reactive species cause direct oxidative damage to fungal cell walls and membranes, disrupting cellular integrity and leading to rapid pathogen elimination. Additionally, the generated reactive species interfere with cellular respiration by interacting with mitochondrial components, effectively compromising the energy metabolism essential for fungal growth and reproduction. The therapy also induces DNA damage through hydroxyl radical formation, preventing cellular replication and repair mechanisms. Furthermore, protein denaturation and enzyme inactivation occur as a result of oxidative stress, disrupting essential metabolic pathways within the dermatophyte cells. A key advantage of CAP-A therapy is its selective targeting mechanism, which preferentially affects pathogenic microorganisms while preserving healthy tissue due to the differential antioxidant capacity between infected and healthy cells.

The treatment protocol was as follows:

- Frequency: 2 times daily.
- Duration: each treatment session consisted of two 3-min fogging cycles.
- Treatment period: 12 consecutive days.
- Application method: non-contact, aerosol delivery via tube.
- Pre-treatment: standard wound monitoring without immediate interventions.
- Post-treatment: standard wound monitoring without immediate interventions.
- Quality control: consistent operator throughout study period.
- No concurrent medications were administered.

To ensure consistent and reproducible treatment outcomes, comprehensive quality control measures were implemented throughout the CAP-A therapy protocol. All operators underwent standardized training to minimize inter-operator variability and ensure proper technique application. These and more standardization protocols are essential for maintaining treatment consistency and enabling reproducible results, while also establishing a foundation for potential multi-centre studies and clinical implementation guidelines.

## 3 Results

**Figure 4:**
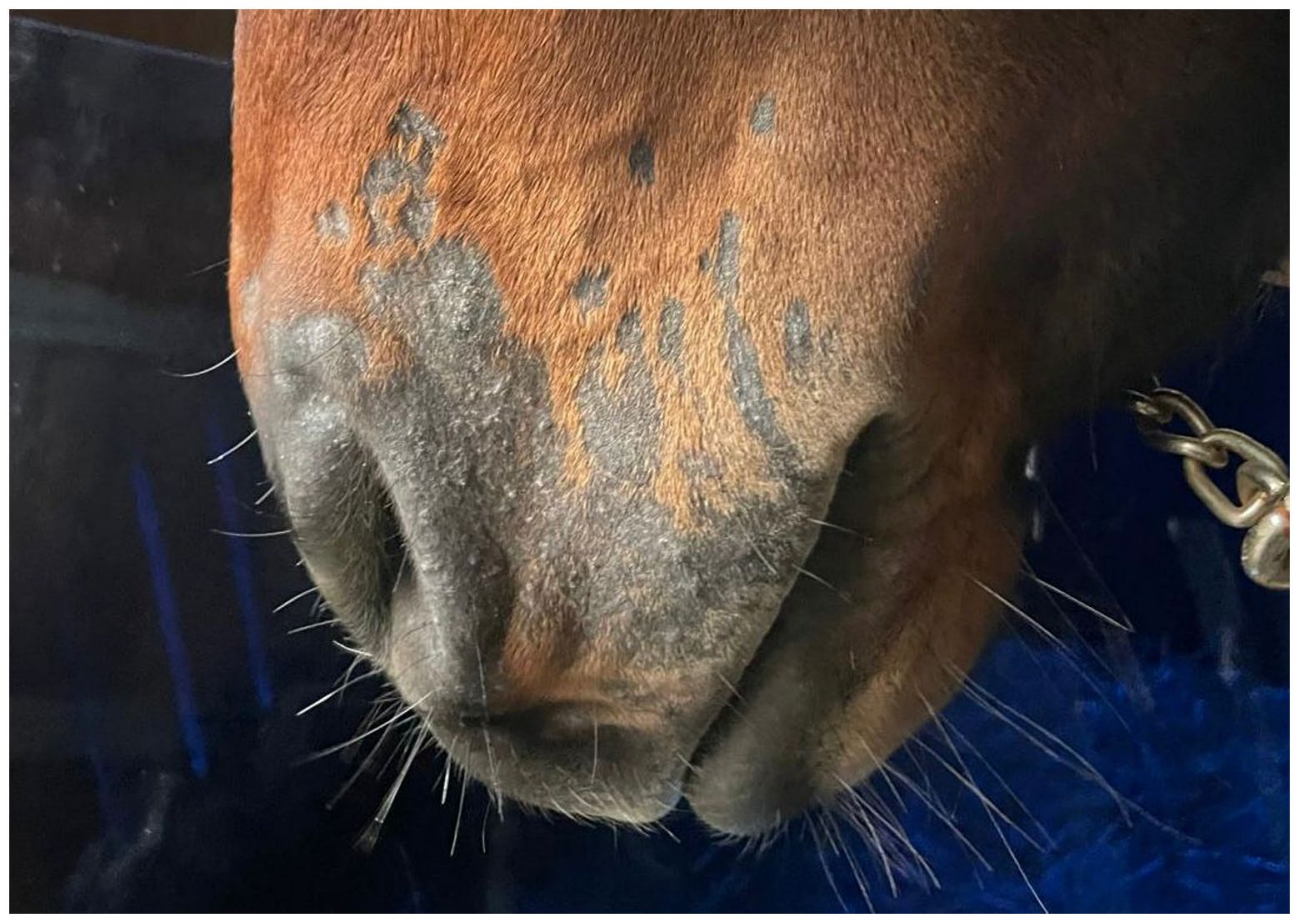
PostTreatment_Lateral.

**Figure 5:**
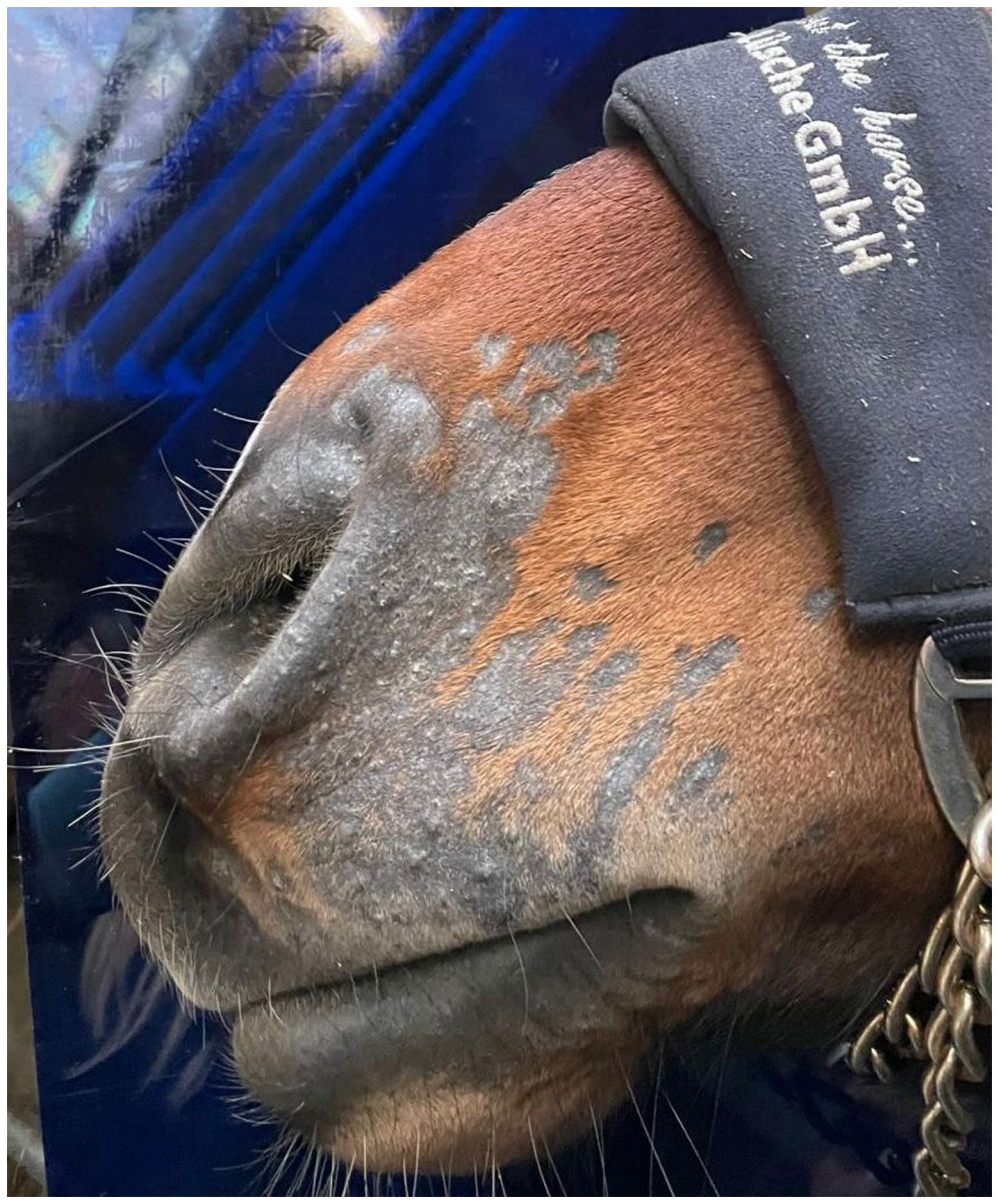
PostTreatment_Frontal.

**Figure 6:**
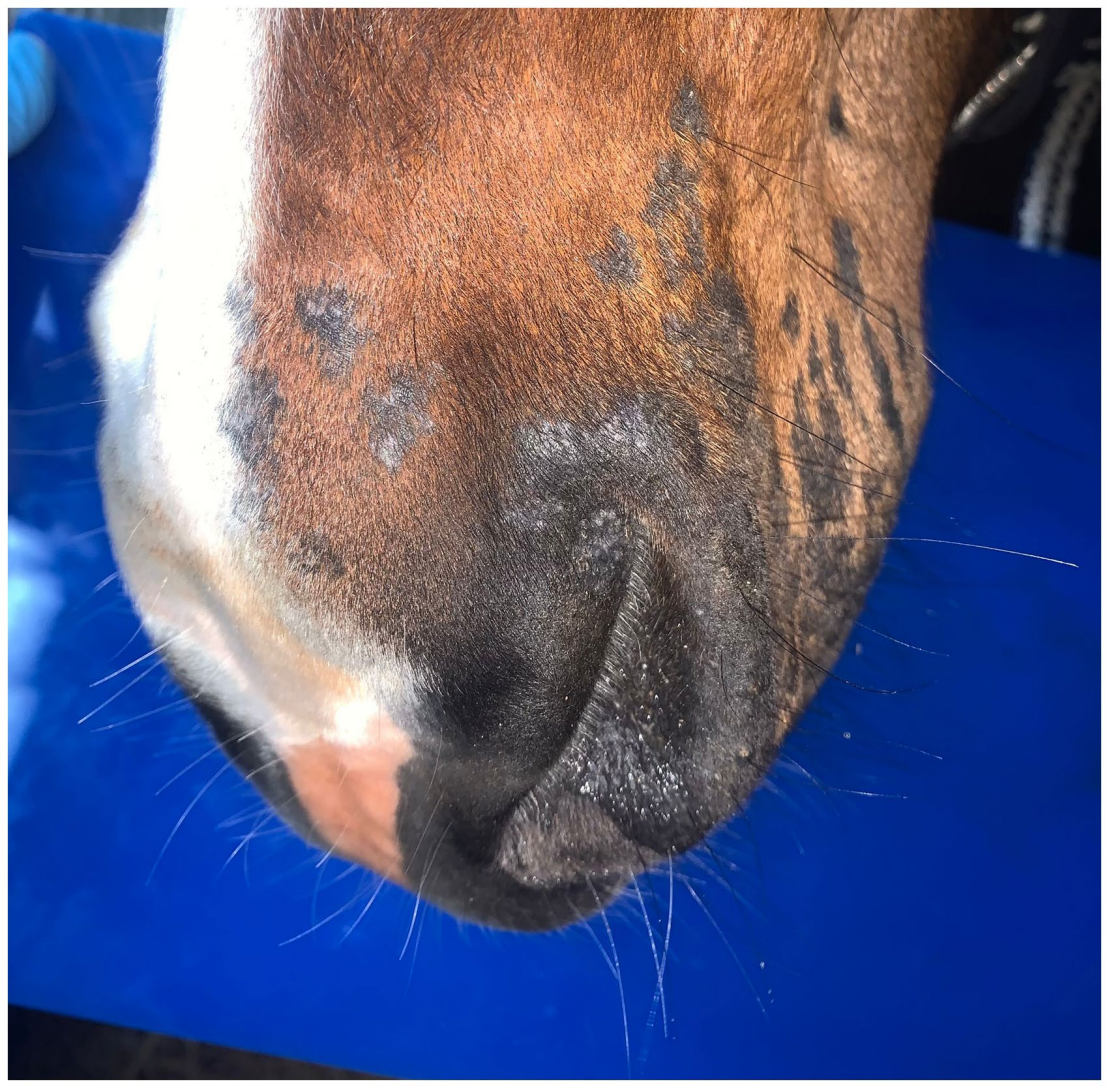
PostTreatment_Oblique.

Post-treatment microbiological analysis showed complete elimination of dermatophyte infection. The presence of normal skin flora indicated restoration of a healthy skin microbiome. Photographic documentation demonstrated progressive clinical improvement (Figures 4–6). The patient showed excellent tolerance to the treatment with no adverse local or systemic reactions and no evidence of pain or discomfort. By day 30 after the beginning of the 12-day treatment course, dermatophyte infection was no longer detectable. No further evidence of recurrence or infection at any other site was noted.

This single case study presents several important limitations that must be considered when interpreting the results. The sample size of *N* = 1 provides no statistical power for efficacy determination and cannot establish population-wide treatment effects, while individual variations in treatment response remain unknown and no possibility exists to identify predictive factors for treatment success. The first findings from this case requiring validation through adequately powered, randomized controlled trials with diverse equine populations and extended follow-up periods.

## 4 Discussion

This case demonstrates superior treatment outcomes compared to conventional antifungal therapy. While traditional topical and systemic antifungals typically require 4–8 weeks for complete resolution, CAP-A achieved clinical and microbiological cure within 12 days, representing a 75% reduction in treatment duration. The complete absence of recurrence during follow-up contrasts with reported recurrence rates of 15–30% for conventional therapies. Unlike systemic antifungals that carry risks of gastrointestinal upset and hepatotoxicity, CAP-A demonstrated no adverse effects while maintaining excellent patient compliance due to its non-invasive, pain-free application.

Cost analysis reveals that CAP-A treatment represents an economically viable alternative to conventional dermatophyte therapy. When considering total treatment costs including veterinary visits, medication expenses, and potential retreatment requirements, CAP-A demonstrates comparable overall costs to traditional antifungal approaches. The shortened treatment duration reduces veterinary visits and monitoring appointments, while higher success rates lower the likelihood of treatment failure requiring alternative therapies. The elimination of systemic medication monitoring removes blood work and hepatic function testing costs, while the simplified treatment protocol reduces labor and handling time.

Zoophilic *Trichophyton* species include *Trichophyton equinum, T. bullosum,* members of the *T. mentagrophytes* complex*, T. simii*, and *T. verrucosum*. These organisms are opportunistic pathogens and most have some zoonotic implications. The most common form occurring in European horses is *T. equinum* var. *equinum* (1–3).

CAP-A treatment is non-specific in terms of its effects, i.e., it targets all microorganisms that respond to high ROS concentrations and might therefore be applicable to a number of other problematic bacterial or fungal skin diseases. It has been successfully used in the treatment of chronic wounds in the past (7–9) and also for the disinfection of surfaces (5, 6, 10). To our knowledge, this case report presents the first documented use of CAP-A as a successful standalone treatment for equine dermatophytosis. Some fungal infections common in horses such as Pythiosus (*Pythium insidiosum*) and Basidiobolomycosis (*Basiodiobollus coronata*) are extremely challenging (2). It should be tested in a prospective follow-up study with a larger sample size whether this method shows efficacy in these cases, which might lead to more effective treatment than the current surgical management that is of limited efficacy and very invasive.

By delivering locally the physical mechanism of solubility and displacement effect in combination with concentrations of ROS, the technology selectively targets microorganisms and host tissue damaged by the infection, while preserving healthy tissue. It represents a potentially significant advantage over conventional treatments. The apparent absence of any pain or discomfort and the ease of delivery suggests superior ease of use in the clinical setting compared to traditional antifungal therapies.

Based on these limitations, comprehensive recommendations for clinical practice should be developed, including consideration of CAP-A therapy as investigational until validation through appropriately powered, randomized controlled trials, identification of suitable candidates such as horses with localized dermatophytosis or those with intolerances to conventional treatments, and establishment of contraindications for pregnant mares and horses with pacemakers as precautionary measures. Future research priorities should encompass randomized controlled trials, multi-center studies across different climates and horse populations, dose–response studies to optimize treatment protocols, and long-term follow-up of 6–12 months to assess recurrence rates.

## Data Availability

The raw data supporting the conclusions of this article will be made available by the authors, without undue reservation.

## References

[1] Scott DW Miller WH. Equine dermatology. 1st ed. Amsterdam, Netherlands: Elsevier. (2003) p. 261–320.

[2] Knottenbelt DC. Pascoe’s principles and practice of equine dermatology. 2nd ed. Philadelphia, USA: Saunders Ltd. (2009). p. 161–185.

[3] Cafarchia C Figueredo LA Otranto D. Fungal diseases of horses. Vet Microbiol. (2013) 167:215–34. doi: 10.1016/j.vetmic.2013.01.015 23428378

[4] Mueller RS. Virale, bakterielle oder pilzbedingte Hautinfektionen beim Pferd. Pferde Spiegel. (2013) 16:106–13. doi: 10.1055/s-0033-1350690

[5] Schmelz U Schaal T Hämmerle G Tischendorf T. The further development of cold plasma technology: the effectiveness of a contactless, indirect atmospheric cold plasma method for germ reduction on surfaces in vitro and in vivo. medRxiv. (2024). doi: 10.1101/2024.10.12.24315382v1 PMC12489233 41048512

[6] Schaal T Schmelz U. Plasma disinfection procedures for surfaces in emergency service vehicles: a field trial at the German red cross. Sci Rep. (2023) 13:20737. doi: 10.1038/s41598-023-47759-5 38007589 PMC10676353

[7] Matzkeit N Schulz L Schleusser S Jensen JO Stang FH Mailaender P. Cold atmospheric plasma improves cutaneous microcirculation in standardized acute wounds: results of a controlled, prospective cohort study. Microvasc Res. (2021) 138:104211. doi: 10.1016/j.mvr.2021.104211, PMID: 34144075

[8] Schleusser S Schulz L Song J Deichmann H Griesmann AC Stang FH. A single application of cold atmospheric plasma (CAP) improves blood flow parameters in chronic wounds. Microcirculation. (2022) 29:e12754. doi: 10.1111/micc.12754 35218286

[9] Jensen JO Schulz L Schleusser S Matzkeit N Stang FH Mailaender P. The repetitive application of cold atmospheric plasma (CAP) improves microcirculation parameters in chronic wounds. Microvasc Res. (2021) 138:104220. doi: 10.1016/j.mvr.2021.104220 34216601

[10] Schmelz U Schaal T Pitten FA Tischendorf T. New approaches to disinfection of thermolabile medical devices using an indirect method with cold atmospheric plasma-aerosol. Sci Rep. (2025) 15:19311. doi: 10.1038/s41598-025-03364-2 40456797 PMC12130244

